# 3D Platform for Coupling Coefficient Evaluation of an Inductive Power Transfer Systems

**DOI:** 10.3390/s22041445

**Published:** 2022-02-13

**Authors:** Jure Domajnko, Miro Milanovič, Nataša Prosen

**Affiliations:** Faculty of Electrical Engineering and Computer Science, University of Maribor, 2000 Maribor, Slovenia; miro.milanovic@um.si (M.M.); natasa.prosen@um.si (N.P.)

**Keywords:** IPT, coupling coefficient, inductance measurement, IPT coil design

## Abstract

This paper presents a custom-made, computer-connected, and controlled 3D platform that enables the evaluation of the coupling coefficient between the transmitter and receiver coil parts of an inductive wireless power transfer (IPT) system. The platform includes a computer application, a 3D positioning mechanism, and an inductance measurement circuit. The positioning mechanism moves the coils to the point in 3D space, and the inductance circuit measures the mutual inductance between the transmitter and the receiver coil. The measured value can be used to calculate the coupling coefficient between the transmitter and the receiver coil. The data are sent to the computer for further visualisation. The transmitter and the receiver coil can be evaluated by measuring the coupling coefficient between them in multiple points in space. Measurements performed with the platform can be used in the design and evaluation phases of inductive wireless power transfer systems and to extrapolate the polynomial function of the coupling coefficient in relation to the distance between coils or their misalignment.

## 1. Introduction

With the rise in the popularity of electric cars and vehicles, new methods of charging their on-board batteries are being proposed and investigated. One such and evermore popular method is wireless charging, especially in the field of electric vehicles [1,2,3,4]. Because of this, wireless power transfer can be used in medical devices, such as circulatory support devices and other medical implants [5,6]. This removes the need for bulky, heavy, and expensive charging cables and connectors. It is also more robust and resistant to environmental influences. However, there are also some drawbacks, the main result of which is the lowered system efficiency and increased system complexity, therefore resulting in a higher initial price and higher maintenance costs.

The most popular method for wireless charging is inductive wireless power transfer (IPT) [4]. The energy is transferred trough a magnetic field that is generated by a transmitter coil and is picked up by a receiver coil. The IPT system usually consists of one transmitter and one receiver coil that are loosely coupled through an air gap. The efficiency of inductive wireless power transfer is usually low because of the weak coupling coefficient. This problem can be overcome by increasing the system frequency using the resonant inductive wireless power transfer method [7,8]. To transfer the maximum possible power compensation, circuits must be added to the transmitter and receiver coils. The coils resonate with their respective compensation at a specific frequency, called the resonant frequency [9]. The resonant frequency is one of the most important design parameters. Most wireless power transfer systems operate with a fixed frequency, but there are also some systems that operate with a variable resonant frequency. Which resonant frequency is used depends on the application of the wireless power system, wireless charging standards, size of the transfer coils, and the power of the system [10,11].

Because of the increased system complexity, more thought, research, and resources must be put into its development and testing. One of the possibilities to optimise and increase the efficiency of IPT systems is to optimise the shape and size of the transmitter and the receiver coil [12,13,14,15]. The optimisation is usually performed using EM simulation software, and the measurements are usually performed manually.

The platform described in this paper can be used in the design and evaluation phases, especially when designing receivers and the transmitter coils. The platform can be used to measure the mutual inductance and coupling coefficient between one transmitter and one receiver coil. The measurements can be used to design coils with a higher coupling coefficient and better misalignment tolerances. A measured coupling coefficient can also be used to optimize the control algorithms for IPT systems to increase power transfer efficiency.

The measurement system can be used as a replacement for the coupling coefficient calculation using an electromagnetic (EM) simulation software such as Ansys Maxwell or Comsol. Simulation software requires a long amount of time to calculate the coupling coefficient between the transmitter and the receiver coil at multiple points. On the other hand, the proposed measurement platform only requires a couple of minutes, depending on the measuring step resolution and the size of the scan area. Compared to EM simulation software, the proposed measurement platform is more complex and requires additional equipment, which may lead to additional costs. On the other hand, the measurement system has been designed in a modular way and can be used to test IPT coils of different shapes and sizes and operating at different frequencies, which could justify the initial investment in the system.

The paper is organised as follows: A detailed description of the testing platform is presented in Section 2. Section 3 describes the inductance measurement method the measurement circuit is based on. The implementation of the measurement system, including the additional circuit analysis, is presented in detail in Section 4. Section 5 presents the measurement results of a few different transfer coils. Section 6 provides a discussion of the results, which measured with the system. Section 7 is the final section of the paper and contains the conclusion.

## 2. Platform Description

The coupling coefficient evaluation platform was designed to be modular. The platform can therefore be used to measure the IPT transfer coils with different shapes and sizes. The frequency at which the transfer coils are evaluated is determined by the application. The measurement system was designed to measure the inductance at a frequency under 150 kHz. The self-inductance of the transmitter and the receiver coil usually range from tens to hundreds of microhenry, and the coupling coefficient is usually below 0.5.

The platform used for measuring the coupling coefficient between the transmitter and the receiver coil consists of three parts:

- A 3D positioning mechanism;
- An inductance measurement circuit;
- A control application.

The proposed system enables the automated measurement of the coupling coefficient between the transmitter and the receiver coil. The 3D positioning mechanism and the inductance measurement circuit are connected to the PC and interact with the control application. Using the application, the user can define the initial parameters of the coupling coefficient measurements. Such parameters are the type of measurement, the starting and ending positions, and the resolution of the measurement.

The measurement system supports two types of measurements: Measurements on the *z* axis only and measurement in the *x*-*y* plane. Measurements in the *z* axis can be used to measure the impact of the distance between the transmitter and the receiver coil on the coupling coefficient. The impact of the distance between the transmitter and the receiver coil is important when the IPT is designed for different transfer distances. The measurement in the *x*-*y* plane can be used to measure the impact of the misalignment between the coils on the coupling coefficient.

Usually, the air gap between the transmitter and the receiver coil is fixed between a couple of millimetres and a couple of decimetres, depending on the application. Therefore, the coil misalignment tolerance is more important than the z distance tolerance. Therefore, the system can be used to test the transmitter and the receiver coil misalignment tolerance at different coil distances.

### 2.1. The Positioning Mechanism

**Figure 1:**
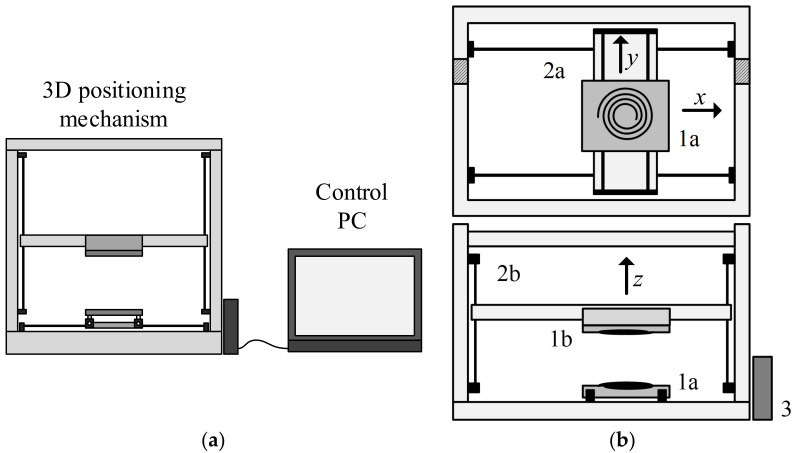
3D positioning mechanism: (**a**) system description; (**b**) 3D positioning platform description.

The positioning mechanism is made of an aluminium body or chassis with three degrees of freedom (3DOF), meaning that it can move in a 3D space. The mechanism is used to position the transmitter and receiver in the 3D space. The mechanism is presented in Figure 1a,b. Figure 1a presents the basic structure of the positioning mechanism, connected to the PC. Figure 1b presents a detailed description of the parts comprising the positioning mechanism. The 3D-positioning chassis mechanism consists of two parts: a top part and a bottom part. The bottom part of this chassis includes a movable platform on sliders (1a), which can then be positioned in both the *x* and *y* directions using toothed belts that are powered by two stepper motors (2a).

The top part includes a platform (1b) that can be moved in the z direction using a threaded shaft and additional stepper motor (2b). Therefore, the whole mechanism is powered by three stepper motors. The transmitter coil is attached to the bottom platform and the receiver coil is attached to the top platform.

The stepper motors are connected to the driving circuits of the stepper motor, which position the system based on signals from the main controller mechanism (3). This main controller is based on the open-source Arduino platform. The controller is connected to a PC via a virtual universal asynchronous receiver-transmitter (virtual UART) port. The controller receives and interprets the instructions sets sent from the PC side of the system and then generates appropriate pulse-width modulated signals (PWM) for the stepper motor drivers. The driving circuits send signals to the stepper motor, which then moves the platforms according to the instructions received. There are no position sensors on the platform axes. Everything is calculated based on the initial reference position of the mechanism. The reference position is determined after the power up process using mechanical limit switches.

### 2.2. Inductance Measurement Circuit

The inductance measurement circuit is used to measure the self and mutual inductance between the transmitter and the receiver coil. The mutual inductance between the transmitter and the receiver coil is calculated from the inductance of the transmitter and the receiver coil connected in series. The measurement circuit consists of two parts. The first part is the switching matrix. The second part is the inductance measurement circuit. The switching matrix consists of relay switches that enable measurements of the inductance of different transmitter and receiver coil configurations and is required for mutual inductance and coupling coefficient calculation.

The inductance measurement circuit is based on the auto-balancing bridge method [16,17] using an operational amplifier. The impedance of the inductor is calculated based on the gain of the amplifier circuit. The measurement circuit requires an external sinusoidal function generator that defines the frequency and amplitude of the voltage the measurement is performed under. The voltage at the input and output of the auto-balancing bridge is alternating current (AC) voltage. Therefore, the inductance measurement circuit also includes a peak detector to measure the amplitude of the input and output signals of the measurement circuit.

The direct current (DC) voltage is measured using the analog-to-digital converter (ADC) of the microcontroller. The resonant frequency of the IPT system can be chosen as the frequency for the input reference AC signal. In case of the IPT for automotive applications, frequencies between 80 kHz and 90 kHz can be chosen, according to [18]. In the case of this paper, the measurements were performed at 86 kHz.

The microcontroller is also used to convert the voltage and gain inductance. The inductance value is the sent via USB link to the computer application, which interacts with the measurement circuit.

### 2.3. Control Application

A Windows application was developed to interact with the position platform and high frequency inverter. It was written in C#. In addition to the application, the computer requires at least one free USB port. The application must be connected to the platform with the correct virtual serial port settings. The virtual seral port connects the positioning mechanism controller and high-frequency inverter controller.

The position mechanism receives and executes text-based commands in the G code instruction format. This is a widely used instruction command language for computer-controlled devices (CNC). The positioning mechanism of can be classified as a CNC device. G code instructions are mainly used for the positioning of the platforms using the stepper motors. A typical line of G code includes the direction of the movement, the relative position of the movement in regard to the reference point, and movement speed. Movement is executed one by one, in sequence.

The application serves as a user interface for the measurement platform. A user can define the type of measurement, step resolution, and the start and the end positions of the measurement.

## 3. The Coupling Coefficient Measurement

### 3.1. The Coupling Coefficient Measurement Method

To evaluate the coupling coefficient between the transmitter and the receiver coil, the platform includes a coupling coefficient measurement circuit. The 3D positioning mechanism moves the coils into the designated position. The measurement circuit then measures the coupling coefficient between the transmitter and the receiver coil. The measurement is based on a well-known method for measuring the inductance of series-connected coils [19]. Coils that are connected in series can have positive or negative mutual inductance. In the case of cumulative mutual inductance, the measured inductance can be expressed with:

(1) $$L_{X1}=L_{T}+L_{R}+2M$$

where *L_X_*_1_ is the measured series inductance in the case of positive mutual inductance, *L_T_* is the self-inductance of the transmitter coil, *L_R_* is the self-inductance of the receiver coil, and *M* is the mutual inductance between the two coils.

When the coils are connected differentially, the measured inductance can be expressed as

(2) $$L_{X2}=L_{T}+L_{R}-2M$$

where *L_X_*_2_ is the measured series inductance in the case of negative mutual inductance. The mutual inductance between the coils can be calculated by joining Equations (1) and (2) in

(3) $$M=\frac{L_{X1}-L_{X2}}{4}$$

The coupling coefficient between the transmitter and the receiver coil can be calculated from the measured mutual inductance and self-inductances of the transmitter and the receiver coils using

(4) $$k=\frac{M}{\sqrt{L_{T}L_{R}}}$$

where *k* is the coupling coefficient between the transmitter and the receiver coil. Therefore, for coupling coefficient evaluation, the measurement system must evaluate four different inductances: the self-inductances of the transmitter coil *L_T_* and receiver coil *L_R_* and series inductances *L_X_*_1_ and *L_X_*_2,_ allowing the coupling coefficient to be calculated.

### 3.2. The Coupling Coefficient Measurement Circuit

**Figure 2:**
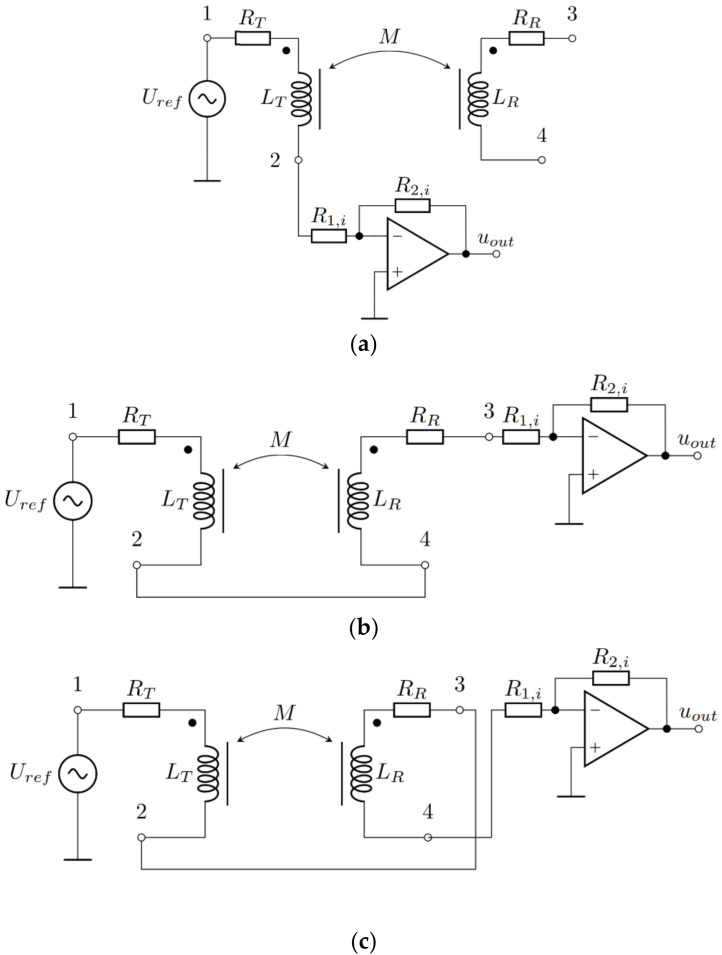
Measurement of the transmitter and receiver coil inductances in different configurations; (**a**) transmitter coil self-inductance; (**b**) series inductance in the case of negative mutual inductance; (**c**) series inductance in the case of positive mutual inductance.

As described before, the coupling coefficient measurement circuit is based on the inductance measurement circuit. The circuit consists of two parts. The first part is the switching part, which includes the relays that connect the transmitter and receiver coil on the inductance measurement circuit in different configurations. The second part is an inductance measuring circuit, which measures the inductance of the transfer coils, which are connected to the input of the switching part of the measurement circuit. The circuit configurations required for mutual inductance and the coupling coefficient measurements are presented in Figure 2a–c. Figure 2a presents the configuration for the measurement of the self-inductance *L_T_*. In this case, only the transmitter coil is connected to the measurement circuit. On a similar principle, the self-inductance of the receiver coil can be measured. The measurement of the series inductance in the case of negative mutual inductance is presented in Figure 2b, and the measurement of the series inductance in the case of positive mutual inductance is presented in Figure 2c.

**Figure 3:**
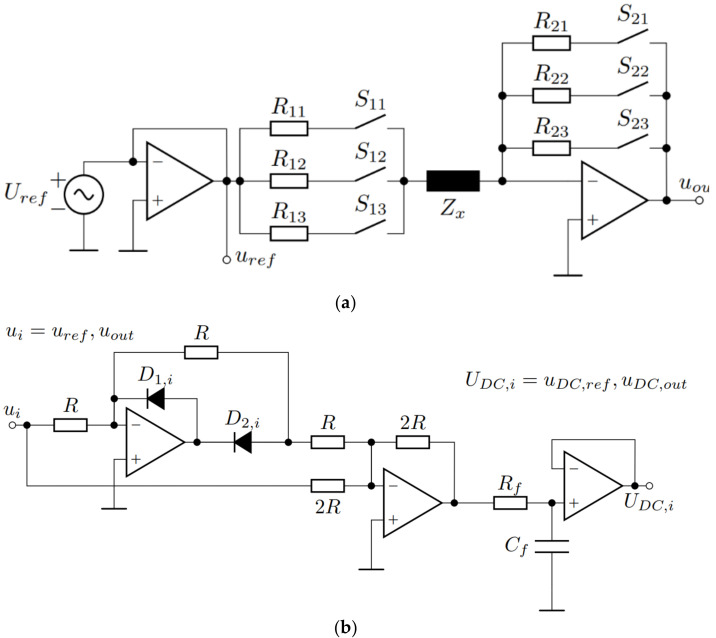
Inductance measurement circuit. (**a**) Auto-balancing bridge. (**b**) Precision rectifier with RC filter for *u**_ref_*** and *u**_out_*** measurement.

The switching part of the coupling coefficient measurement circuit is connected to the amplifier in the inverting configuration, which is also presented in Figure 2. The inverting amplifier forms an auto-balancing bridge, which serves as an inductance measurement circuit. The circuit is shown in more detail in Figure 3. Figure 3a presents the auto-balancing bridge, and Figure 3b presents the precision rectifier with an RC filter. The auto-balancing bridge is used for the inductance measurements, and the precision rectifier is used to convert the AC voltages from the auto-balancing bridge to the DC voltages, which can be measured using a microcontroller.

The input of the auto-balancing bridge circuit is connected to the external function generator, which generates a sinusoidal voltage with the frequency ω*_ref_*. At the output of the circuit is the AC voltage *u_out_*, which is the amplified input voltage. The gain of the circuit is defined with resistors and unknown impedance. The circuit includes the resistances *R*_11_, *R*_12_, and *R*_13_ at the input of the operational amplifier, and the resistances *R*_21_, *R*_22,_ and *R*_23_ in the negative feedback loop. The resistors are connected to the operational amplifier using signal relays.

Gains in the circuit are defined based on which switches are turned on. The circuit is used to measure the unknown impedance *Z_x_*, which can be expressed with

(5) $$Z_{x}=R_{x}+j\omega_{ref}L_{x}$$

where *Z_x_* is the unknown impedance, *R_x_* is the real part, and *L_x_* is the inductive imaginary part of the unknown impedance. The configuration of the coils during unknown impedance Z*_x_* is defined by the input switching portion. If only self-inductances are measured, *Z_x_* takes the form of

(6) $$Z_{x}=R_{T}+j\omega_{ref}L_{T}$$

and

(7) $$Z_{x}=R_{R}+j\omega_{ref}L_{R}$$

When measuring mutual inductance, the *Z_x_* of the series-connected inductors with negative mutual inductance is defined as

(8) $$Z_{x}=R_{T}+R_{R}+j\omega_{ref}L_{X1}$$

Additionally, in the case of positive mutual inductance:

(9) $$Z_{x}=R_{T}+R_{R}+j\omega_{ref}L_{X2}$$

The unknown impedance value impacts the gain of the system. The gains in the measurement circuit are defined using:

(10) $$G_{i}=-\frac{R_{2,i}}{R_{1,i}+Z_{x}}=\frac{U_{out}}{U_{ref}},\;i=1,\;2,\;3\;$$

where *G_i_* is the gains in the measurement circuit, *R*_2,*i*_ is the resistance of the resistor in the negative feedback loop, and *R*_1,*i*_ is the resistance of the resistor in series with the unknown load *Z_x_*. By changing the value or *R*_1,*i*_ and *R*_2,*i*_, the measurement range and sensitivity of the circuit also change. The absolute gains are also defined as a quotient between the amplitude of the output voltage *U_out_* and the amplitude of the input voltage *U_ref_*. By measuring the amplitudes of both voltages, the impedance value, which consists of a series connection between the unknown impedance *Z_x_* and the known resistance *R*_1,*i*_, can be calculated using:

(11) $$\left|Z_{x}+R_{1,i}\right|=\left|R_{x}+j\omega L_{x}+R_{1,i}\right|=\left|\frac{R_{2,i}}{\left(\frac{U_{out}}{U_{ref}}\right)}\right|$$

The unknown impedance *Z_x_* also has a real resistive component. Therefore, the best way to calculate the inductive component *L_x_* is by using the phase angle between the input and the output voltage:

(12) $$L_{x}=\frac{R_{2,i}}{\frac{U_{out}}{U_{ref}}}\cdot \sin\left(\phi \right)$$

where ϕ is the phase angle between the input and the output voltage. The phase angle is measured using the zero-crossing detector and timer peripheral of the digital signal controller enhanced capture (eCAP) unit. It is also important to note that the operational amplifier in the inverting configuration changes phase by −180°.

The resistive component of the unknown impedance can be calculated using:

(13) $$R_{x}=\frac{R_{2,i}}{\frac{U_{out}}{U_{ref}}}\cdot \cos\left(\phi \right)-R_{1,i}$$

Finally, the unknown inductance at the input of the circuit can be calculated from the imaginary component of the unknown impedance and the frequency of the AC reference voltage *f_ref_*:

(14) $$L_{x}=\frac{R_{2,i}}{2\pi f_{ref}\frac{U_{out}}{U_{ref}}}\cdot \sin\left(\phi \right)$$

Voltages *U_out_* and *U_ref_* are measured using a precision rectifier with an RC filter. The circuit converts the AC voltage to DC voltage, where *U_i_* represents the *U_ref_* or *U_out_*, and *U_DC,i_* represents the *U_DC,ref_* or *U_DC,out_*. The DC voltage can be converted to the peak value using the equation

(15) $$U_{i}=\frac{2}{\pi }\cdot U_{DC,i}$$

where *U_i_* is the stand in for *U_ref_* or *U_out_*, which are used to calculate the measured inductance using Equation (14).

## 4. Practical Implementation of the Inductance Measurement Circuit

**Figure 4:**
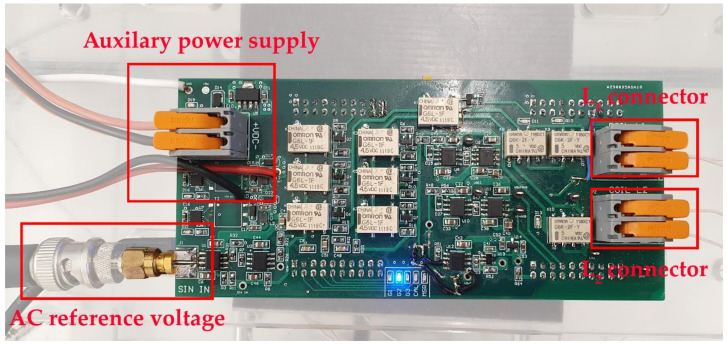
Circuit of the auto-balancing bridge with variable gain.

The circuit of the coupling coefficient measurement circuit is presented in Figure 4. The circuit includes the previously described input switching matrix using relay switches, the auto-balancing bridge with switches for different gain configurations, and additional circuits to convert the AC voltage to DC voltage. The input connectors are marked with red squares.

The circuit has two connectors to connect the transmitter and the receiver coil. To excite the coils with sinusoidal voltage, an external function generator is required, which is connected to the circuit via a Bayonet Neill–Concelman (BNC) connector. The circuit also requires an external auxiliary power supply to power up the microprocessor, operational amplifiers, and relays.

For phase angle measurement, inductance calculation, and communication, a microcontroller (Dallas, TX, USA) development board TI LAUNCHXL-F28379D with a microprocessor TMS320F28379D (Dallas, TX, USA) was used for ADC signal conversion. The amplitude of the input and output voltage was measured using the ADC converter, and the phase angle was measured using the eCAP module used for capturing and measuring the duration of the signal pulses. The measured inductance value was sent to the PC using virtual serial port (virtual UART).

The microcontroller development board also receives data from the control application. The data include information on which switches in the input switching matrix must be open, the gain in the amplifier, and when to start the measurement. When the measurement is completed, the microcontroller returns the data about the inductance in a specific spot. The coils can then be moved to the next point, and the measurement can be performed again.

**Figure 5:**
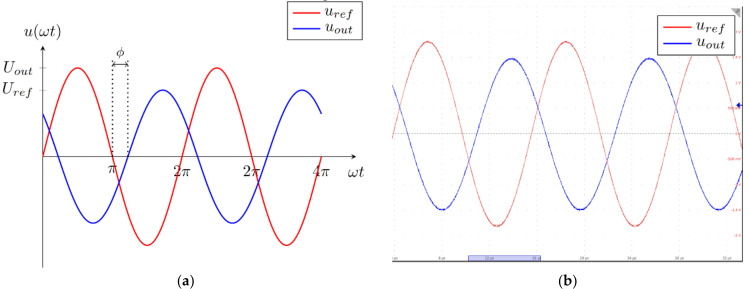
Voltage signals on the auto-balancing bridge. (**a**) Theoretical values, (**b**) measured values.

The theoretical and measured waveform of the input and output voltage on the measurement circuit are presented in Figure 5a,b. Figure 5a presents the theoretical waveforms, where the red signal line represents the input or reference voltage of the measurement circuit, and the blue line represents the output voltage of the measurement circuit. The phase angle between the voltages is denoted as ϕ. Figure 5b presents the input and output signal waveforms as measured on the circuit using an oscilloscope. The input or reference signal is presented with the red signal waveform, and the output signal is presented with the blue signal waveform.

Additional auxiliary circuits measure the amplitude of the input and output voltages and the phase angle ϕ. The microcontroller uses those values to calculate the unknown inductance.

The circuit has a variable gain that is designed for the measurement of different ranges of inductances. The resistors used for the measurements are presented in Table 1. The sampling frequency of the microcontroller ADC was 1 kHz had a 12-bit resolution.

**Figure 6:**
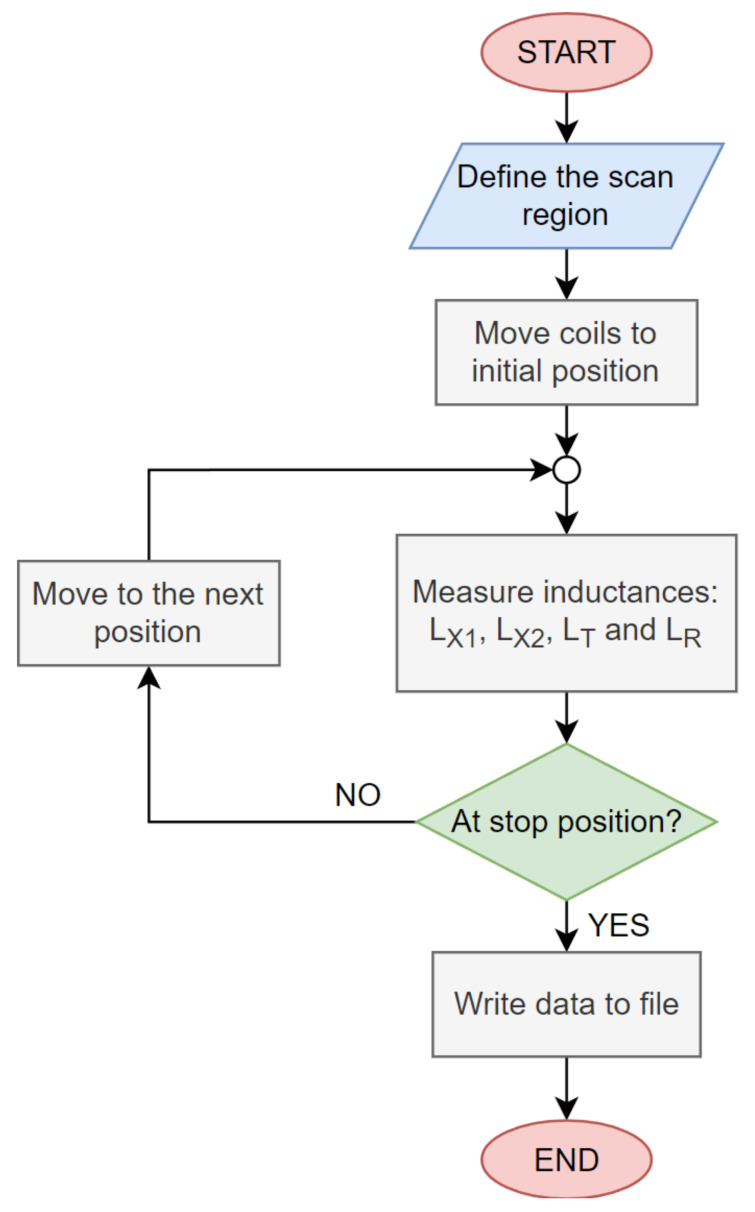
Inductance measurement flowchart.

A flowchart of the inductance measurements is presented in Figure 6. At the start of the automatic measurements, the user defines the type of measurement using the start and the endpoint of the measurement. This defines the scan area. The 3D system then positions the coils in the correct position. At the point in space, the inductance measurement circuit measures values of *L_X_*_1_, *L_X_*_2_, *L_T_*, and *L_R_*, which are then used to calculate the mutual inductance and the coupling coefficient. If the positioning mechanism is not in the end position, then the coils are moved into the next position, and the measurement is repeated. The measurement system stops at the endpoint. The measurement data are then saved to a file and can be further visualised and analysed.

### 4.1. Bandwidth of the Measurement Circuit

The core of the measurement circuit is the auto-balancing bridge circuit, which is basically the operational amplifier in the inverting configuration. The bandwidth of the measurement circuit is therefore determined by the bandwidth of the amplifier circuit.

The amplitude characteristics of the open-loop gain in the operational amplifier *A* can be described with

(16) $$A\left(j\omega \right)=\frac{A_{o}}{1+\frac{j\omega }{\omega_{t}/A_{o}}}$$

where *A_o_* is the open-loop gain at a frequency of 0 rad/s, and ω*_t_* is the cross-over frequency of the operational amplifier when the open-loop gain in the operational amplifier is 0 dB. The resistors *R*_1,*i*_ and *R*_2,*i*_ with unknown impedance *X_L_* form the closed-loop system, which limits the gain. The closed-loop gain in the measurement circuit *G* can therefore be described as:

(17) $$G_{i}\left(j\omega \right)=-\frac{\frac{R_{2,i}}{R_{1,i}+Z_{x}}}{1+\left(1+\frac{R_{2,i}}{R_{1,i}+Z_{x}}\right)\frac{1}{A\left(j\omega \right)}}=-\frac{\frac{R_{2,i}}{R_{1,i}+R_{x}+j\omega L_{x}}}{1+\left(1+\frac{R_{2,i}}{R_{1,i}+R_{x}+j\omega L_{x}}\right)\frac{1}{A\left(j\omega \right)}}$$

where *R*_1,*i*_ and the *R*_2,*i*_ are the gain resistors, and *Z_x_* is the unknown impedance at the input of the circuit. The transfer function can be rewritten as

(18) $$G_{i}\left(j\omega \right)=-\frac{G_{a}}{1+\frac{j\omega }{\omega_{a,i}}}\frac{1}{1+\left(1+\frac{j\omega }{\omega_{a,i}}\right)\frac{j\omega }{\omega_{t}}}$$

where gain *G_a_* and frequency ω*_a,i_* can be described as

(19) $$G_{a,i}=\frac{R_{2,i}}{R_{1,i}+R_{x}}$$

(20) $$\omega_{a,i}\left(j\omega \right)=\frac{R_{1}+R_{x}}{L_{x}}$$

For the chosen amplifier, the open-loop gain was 150 dB and the transition frequency was 1.02 × 10^8^ rad/s. The measurement system operates below the transition frequency of the operational amplifier at 5.47 × 10^5^ rad/s. The frequency-dependent gain of the amplifier therefore does not impact the frequency characteristics of the measurement circuit at the lower frequencies. Therefore, the transfer function of the measurement system can be defined with a first order transfer function as

(21) $$G_{i}\left(j\omega \right)=\frac{G_{a,i}}{1+\frac{j\omega }{\omega_{a,i}}}$$

**Figure 7:**
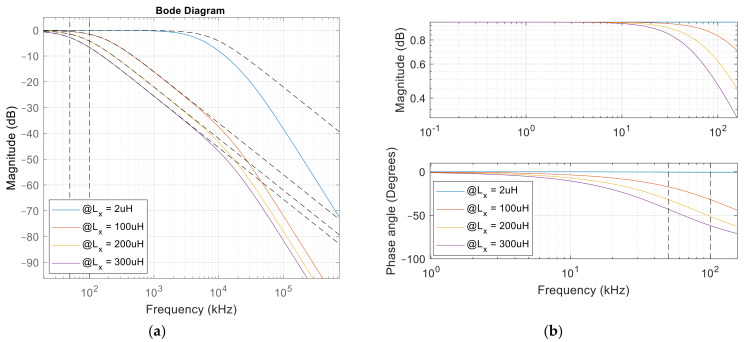
Bode plot of the measurement circuit at different inductances. (**a**) Bode plot of the extended transfer function, (**b**) Bode plot of the simplified transfer function.

The gain in the measurement system is dependent on the unknown impedance *Z_x_*. Therefore, the Bode magnitude plot is dependent on the value Lx and resistance *Z_x_*. Figure 7a presents the Bode plot at four different inductances. The first inductance is 2 μH, marked with the blue line, the second 100 μH, marked with the red line, the third is 200 μH, marked with the yellow line, and the fourth inductance is 300 μH, which is marked with the purple line. The Bode plot using the extended transfer function (17) is marked with full lines, and the Bode plot using the simplified transfer function (21) is marked with dashed lines. At frequencies below 1.0 × 10^7^ rad/s, the simplified transfer function behaves similarly to the extended transfer function. The measurement system operates at 5.47 × 10^5^ rad/s (87 kHz), which is well below the frequency where the simplified transfer function differs from the extended transfer function. The extended transfer function behaves as a second order low-pass filter, and the simplified transfer function behaves similar to the first order low-pass filter. Therefore, Equations (11)–(13) can be used to calculate the unknown inductance from the phase angle and the gain of the circuit.

The bandwidth of the system, measuring inductance 2 μH is 4.82 × 10^7^ rad/s, the bandwidth of the system measuring inductance 100 μH is 8.95 × 10^6^ rad/s, the bandwidth of the system measuring inductance 200 μH is 4.74 × 10^6^ rad/s, and the bandwidth of the system measuring inductance 300 μH is 3.21 × 10^6^ rad/s. The bandwidth of the measurement system therefore decreases at higher inductances.

Figure 7b presents the bode magnitude and phase plot for a simplified transfer function in the measurement range between 50 kHz and 100 kHz. The inductance impacts the gain in the circuit and the phase angle between the input and the output voltages.

### 4.2. Sensitivity Analysis of the Measurement Circuit

The sensitivity analysis of the measurement circuit was performed to test the sensitivity of the circuit, which is dependent on the tolerance of the feedback gain resistors. The sensitivity function used in the analysis can be defined as:

(22) $$S_{x}^{y}=\frac{\partial y}{\partial x}\frac{x}{y}$$

where the component is denoted as *x,* and the circuit parameter is denoted as *y*. In the case of this analysis, the circuit parameter *x* is the closed-loop gain *G* at the frequency ω*_a,i,_* and the circuit parameter is the closed-loop *G_i_* in the absolute form.

(23) $$G_{A}=\left|G\left(j\omega \right)\right|=\frac{G_{a,i}}{\sqrt{1+{\left(\frac{j\omega }{\omega_{a,i}}\right)}^{2}}}$$

From Equation (21) it can be concluded that the absolute value of the closed-loop gain is sensitive to two parameters, gain *G_a,i_*, defined by resistors *R*_1,*i*_, *R*_2,*i*_, and the impedance *Z_i_*, and the closed-loop cut-off frequency ω*_a_*_,*i*_. Using (22) and (23), two sensitivity functions can be defined as

(24) $$S_{G_{i}}^{G_{A}}=\frac{dG_{A}}{dG_{a,i}}\frac{G_{a,i}}{G_{A}}=1$$

(25) $$S_{\omega_{b}}^{G_{A}}=\frac{dG_{A}}{d\omega_{a,i}}\frac{\omega_{a,i}}{G_{A}}=\frac{\omega }{\omega_{a,i}^{2}+\omega^{2}}$$

The closed-loop gain in the measurement circuit depends on the resistances *R*_1,*i*_ and *R*_2,*i*_. In the case of this paper, the tolerances of the resistors were chosen as 0.5%. Therefore, the tolerance of the gain *G_A_* is 1%. According to sensitivity Equation (24), the variation in the gain is

(26) $$\frac{dG_{A}}{G_{A}}=S_{G_{a,i}}^{G_{A}}\frac{dG_{a,i}}{G_{a,i}}=\pm 1\%$$

The variation in the gain, which is dependent on frequency, can be calculated for two different frequencies using Equation (25). The first frequency was chosen as ω = 0 rad/s, and the second frequency was chosen as ω = ω*_a,i_*. This results in the following equations:

(27) $${\left.S_{\omega_{a,i}}^{G_{A}}\right|}_{\omega =0}=0$$

(28) $${\left.S_{\omega_{b,i}}^{G_{A}}\right|}_{\omega =\omega_{a,i}}=\frac{1}{2}$$

From Equations (27) and (28), the variation in the closed-loop gain can be expressed as

(29) $${\left.\frac{dG_{A}}{\omega_{a,i}}\right|}_{\omega =0}=S_{\omega_{b,i}}^{G_{A}}\frac{d\omega_{a,i}}{\omega_{a,i}}=0$$

(30) $${\left.\frac{dG_{A}}{\omega_{a,i}}\right|}_{\omega =\omega_{a,i}}=S_{\omega_{a,i}}^{G_{A}}\frac{d\omega_{a,}{}_{i}}{\omega_{a,i}}=\pm 0.95\%$$

From the sensitivity analysis based upon Equations (26), (29) and (30), it can be concluded that the maximum variation in the closed loop gain is 1%.

## 5. Coupling Coefficient Measurement Results

The proposed measuring platform was used to evaluate the coupling coefficient between the two coils. Two different coil structures were evaluated in this case. The first structure was a classic square spiral coil that placed on a square ferrite pad with the dimensions 100 mm × 100 mm × 6 mm. The second coil structure was a planar DD coil structure generating a directional magnetic field [20,21,22]. The DD coil is composed of two rectangular D coils that are connected in series. Each of the D coils had nine turns and outer dimensions of 50 mm × 100 mm. The measurement system was used to evaluate the coupling coefficient on the *z*-axis and the misalignment tolerance between the coils in different *x*-*y* planes at different *z*-axis distances. Measurements in the *x*-*y* plane were performed at four different *z* distances. The results are presented in the next two subsections.

**Figure 8:**
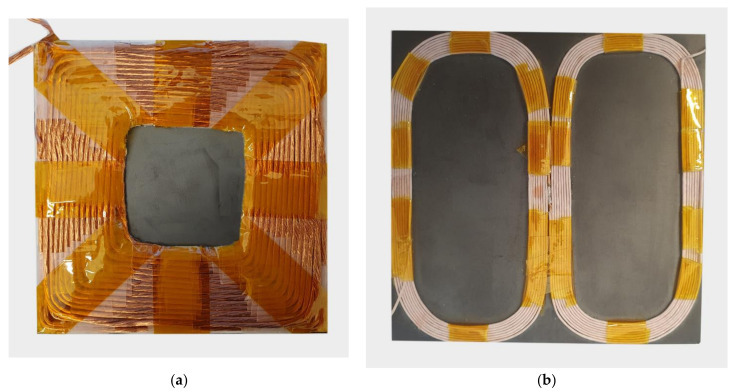
Coils under test. (**a**) Square planar coil, (**b**) planar DD coil.

The coils used in the experimental evaluation are presented in Figure 8. Figure 8a presents a planar spiral coil with a self-inductance of 144.2 μH. Figure 8b presents a planar DD coil with a self-inductance of 45 μH.

### 5.1. Quadrature Planar Coil Measurement Results

**Figure 9:**
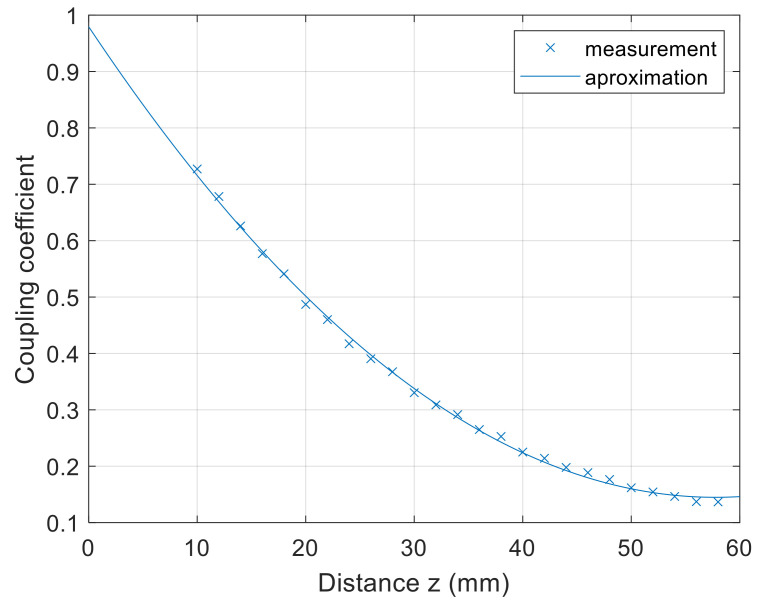
*z*-axis coupling coefficient measurement of planar square coils.

The measurement results of the square spiral coils are presented in the Figures below. Figure 9 presents the coupling coefficient characteristics in the *z*-axis direction. At the 10 mm distance between the coils, the coupling coefficient was around 0.73. When the distance between the coils increased, the coupling coefficient decreased. The minimum measured value between the coils was around 0.15 at the distance of 58 mm.

The coupling coefficient characteristics in the *z* axis can be approximated using the second-order polynomial equation

(31) $$k\left(z\right)=2.5\times 10^{-4}z^{2}-2.889\times 10^{-2}z+0.9795$$

where *k*(*z*) is the coupling coefficient, and *z* is the distance between the coils in mm.

**Figure 10:**
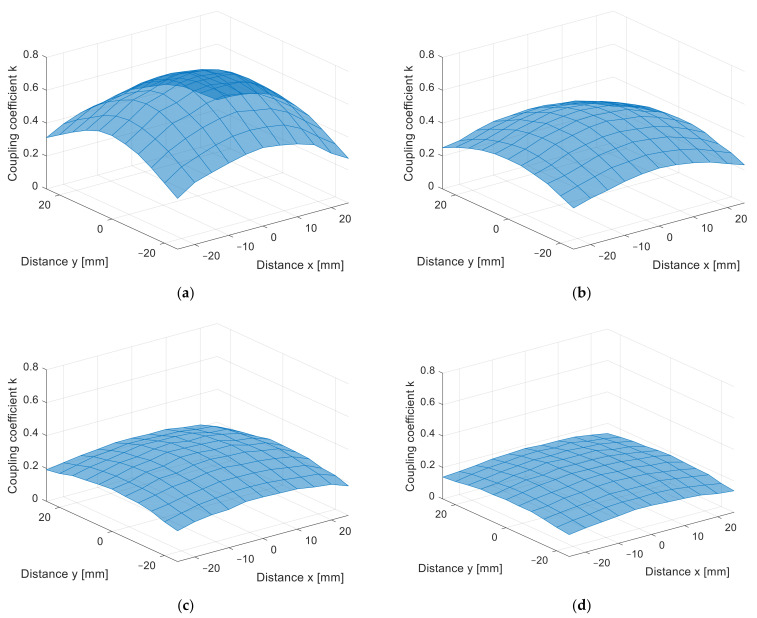
*x*-*y* coupling coefficient measurement of planar square coils (3D surface plot). (**a**) At *z* = 10 mm, (**b**) at *z* = 15 mm, (**c**) at *z* = 20 mm, (**d**) at *z* = 25 mm.

**Figure 11:**
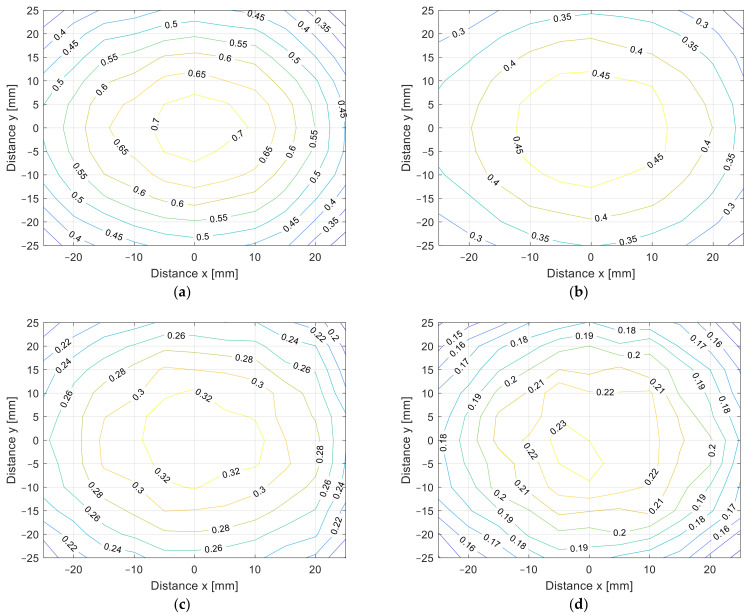
*x*-*y* coupling coefficient measurement of planar square coils (contour plot). (**a**) At *z* = 10 mm, (**b**) at *z* = 15 mm, (**c**) at *z* = 20 mm, (**d**) at *z* = 25 mm.

The misalignment measurement results are presented in Figure 10a–d in 3D surface form and in Figure 11a–d in contour form. The value of the coupling coefficient decreased when the coil was misaligned. The measurements were performed at different distances, from *z* = 10 mm in Figure 10a to *z* = 25 mm in Figure 10d, with 5 mm increments. Naturally, the coupling coefficient is the largest when the distance between the transfer coils is the smallest. The square coils show symmetrical misalignment tolerance. The coupling coefficient decreases with the horizontal misalignment. When the coils were misaligned by 25 mm, the coupling coefficient decreased by more than 60%. This also resulted in lower IPT transfer efficiency and additional unnecessary losses. A similar bad misalignment tolerance can be observed in all of the nonpolar IPT coil topologies.

**Figure 12:**
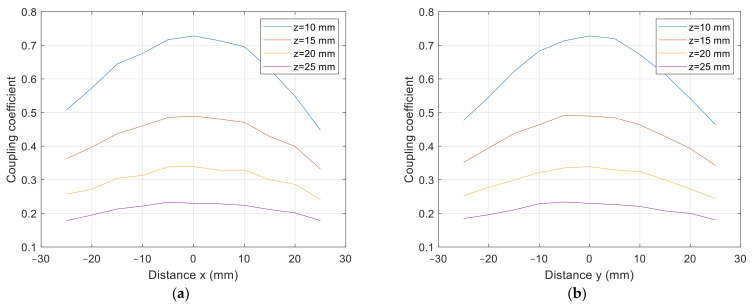
*x*-*y* coupling coefficient measurement of planar square coils. (**a**) At *y* = 0 mm, at (**b**) *x* = 0 mm.

The cross-section of the 3D graph presented in Figure 10 and Figure 11 is presented in Figure 12a,b. Figure 12a presents the coupling coefficient measurement on the *x* axis at *y* = 0 mm, and Figure 12b presents the coupling coefficient measurement on the *y* axis at *x* = 0 mm. From the results, it can be observed that the coupling coefficient decreased symmetrically in both axes.

### 5.2. DD Planar Coil Measurement Results

The main purpose of the directional DD coil is to create better tolerance to misalignment. The DD planar coils were first developed and described in [20,21,22] as an alternative to the classic nonpolar coil structures usually used in a commercial IPT. The DD coil structure is named after its unique shape—the coil is composed of two square or rectangular coils that are connected in series. The DD coil generates a directional magnetic flux along one of the axes—similar to a flux–pipe-type coupler [23,24,25,26]. The main advantage of the DD coil is better misalignment tolerance compared to the nondirectional planar coils.

**Figure 13:**
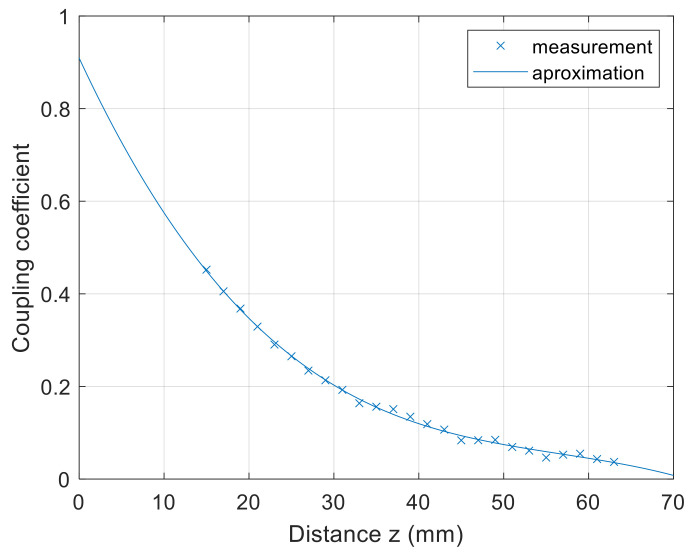
*z*-axis coupling coefficient measurement of planar DD coils.

The results of the directional DD coil measurements in the *z* direction are presented in Figure 13. The coupling coefficient was measured at distances between the 15 and 65 mm in 2 mm increments. The maximum coupling coefficient was 0.45 at 15 mm. Compared to the square coil, the coupling coefficient of the DD coil has a similar characteristic. The overall value was lower, something that was mainly due to the main coil area. The square planar coil has a greater area and larger inductance compared to the DD coil. This results in an overall lower coupling coefficient value at the same distance between the coils.

The coupling coefficient characteristics in the *z* axis can be approximated using the third order polynomial equation:

(32) $$k\left(z\right)=-3.789\times 10^{-6}z^{3}+4.455\times 10^{-4}z^{2}-3.95\times 10^{-2}z+0.9091$$

where *k*(*z*) is the coupling coefficient, and *z* is the distance between the coils in mm.

**Figure 14:**
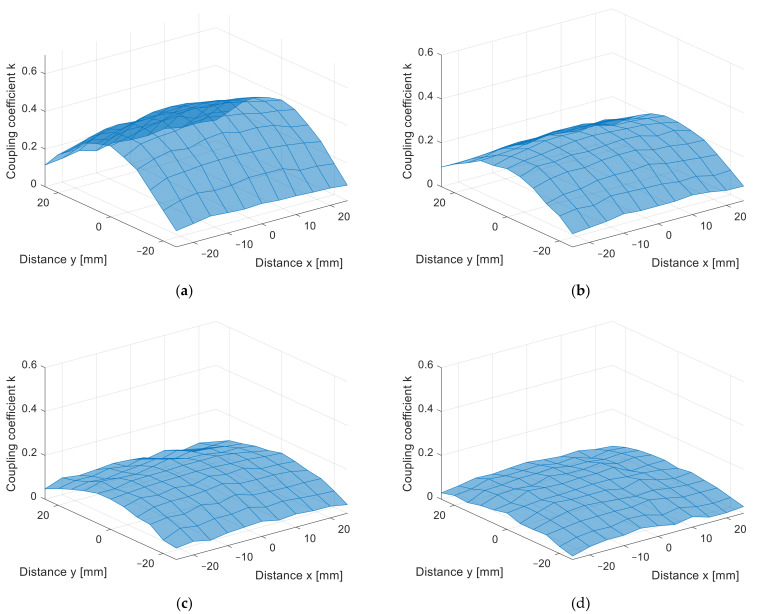
*x*-*y* coupling coefficient measurement of planar DD coils (3D surface plot). (**a**) At *z* = 10 mm, (**b**) at *z* = 15 mm, (**c**) at *z* = 20 mm, (**d**) at *z* = 25 mm.

**Figure 15:**
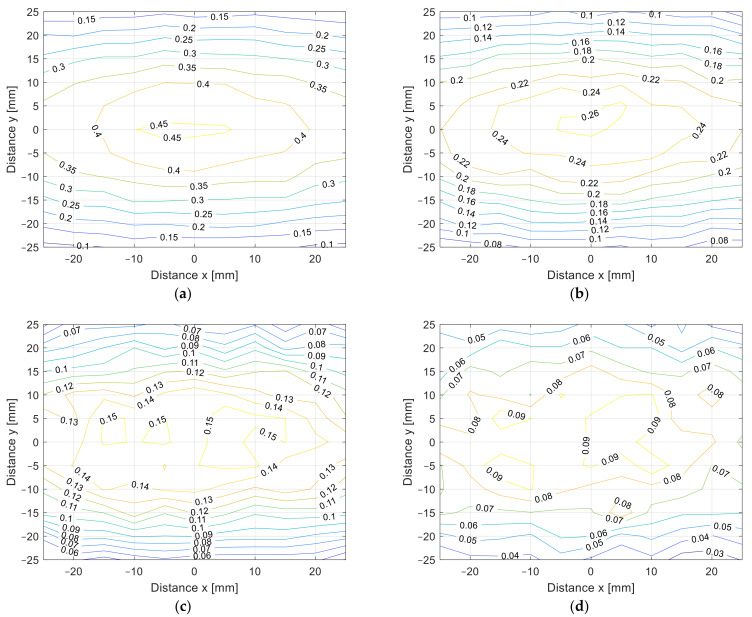
*x*-*y* coupling coefficient measurement of planar DD coils (contour plot). (**a**) At *z* = 10 mm, (**b**) at *z* = 15 mm, (**c**) at *z* = 20 mm, (**d**) at *z* = 25 mm.

From the measurement in the *z* direction, the only difference between the coil structures was the value of the coupling coefficient due to the different coil dimensions. The coupling coefficient still reduced with the distance. The DD coil performed better in the *x*-*y* plane measurements due to the directional magnetic flux. The results of the measurements are presented in Figure 14a–d in 3D surface form and in Figure 15 in contour form. Figure 14a presents the measurements in the *x*-*y* plane at *z* = 10 mm, Figure 14b at *z* = 15 mm, Figure 14c at *z* = 20 mm, and, lastly, Figure 14d presents the measurements at *z* = 25 mm. From the measurements, it can be observed that the coupling coefficient does not reduce symmetrically in the *x* and *y* axes. When the coils are misaligned in the *y* direction, the coupling coefficient reduces drastically when compared to the coil misalignment in the *x* direction. Therefore, DD coils are more suitable for applications when tolerance to misalignment in one direction is possible. In the case of automotive applications, the vertical *z* distance is dependent on the tire pressure, which is more or less constant and is unavoidable. The horizontal *x*-*y* plane misalignment is dependent on the position of the vehicular receiver coil above the transmitter coil of the IPT charger. The DD coils allow for a significantly larger amount misalignment in one horizontal direction compared to the square planar transfer coils and are therefore more usable.

**Figure 16:**
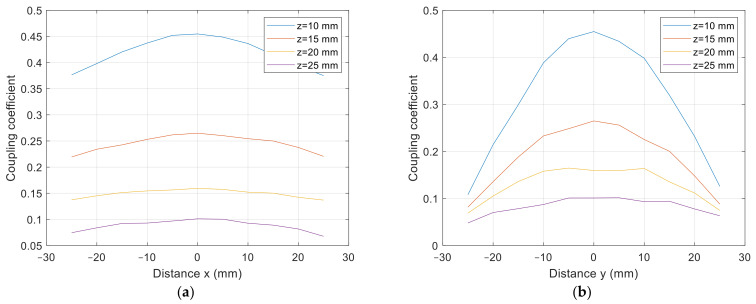
*x*-*y* coupling coefficient measurement of planar DD coils. (**a**) At *y* = 0 mm, at (**b**) *x* = 0 mm.

The cross-section of the 3D graph presented in Figure 14 and Figure 15 is presented in Figure 16a,b. Figure 16a presents the coupling coefficient measurement in the *x* axis at *y* = 0 mm, and Figure 16b presents the coupling coefficient measurement in the *y* axis at *x* = 0 mm. From the results it can be observed that the coupling coefficient does not decrease symmetrically. The coupling coefficient of the directional DD coil is less tolerant to the misalignment on the y axis than misalignment on the *x* axis.

**Figure 17:**
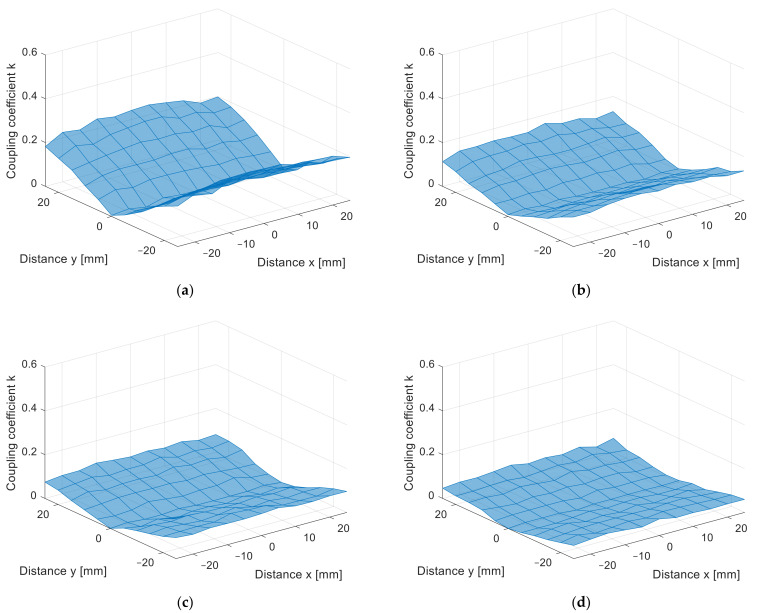
*x*-*y* coupling coefficient measurement between the DD coil and square coil (3D surface plot). (**a**) At *z* = 10 mm, (**b**) at *z* = 15 mm, (**c**) at *z* = 20 mm, (**d**) at *z* = 25 mm.

**Figure 18:**
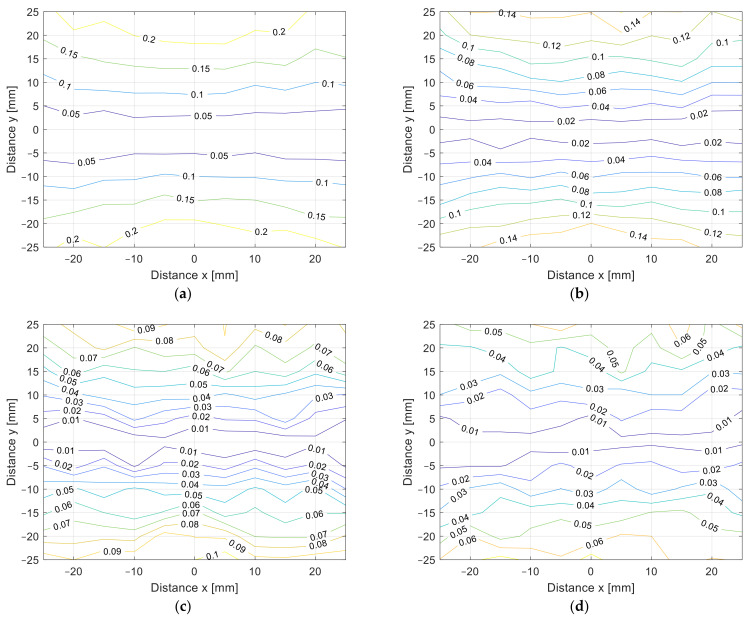
*x*-*y* coupling coefficient measurement between the DD coil and square coil (contour plot). (**a**) At *z* = 10 mm, (**b**) At *z* = 15 mm, (**c**) At *z* = 20 mm, (**d**) *z* = 25 mm.

Figure 17 presents the results of the coupling coefficient measurement between the square planar coil and one DD coil in 3D surface form. The contour form is presented in Figure 18. The measurements were performed at four different *z* distances. Because the DD coil generates the directional field and the square coil generates the nondirectional field, the coupling coefficient can reduce to the zero. This happens when coils are aligned perfectly. From the results, it can be observed that the coils were aligned perfectly when the coupling coefficient was zero. The DD coil generates a directional magnetic field along the *x*-axis. Therefore, the combination of the DD coil and square coil can only be used when the coils are misaligned.

## 6. Discussion

The proposed measurement platform was used to test different IPT coil configurations and combinations. The results can be used to optimise different IPT systems during the design and testing phase. The platform can be used to test the coupling coefficient and mutual inductance between the transmitter and receiver coil in the *z*-direction and in the *x*-*y* plane at different distances between the coils. The measurements in the *z*-direction can help designers determine the maximum and minimum *z* distance between the transmitter and the receiver coil. On the other hand, measurements in the *x*-*y* plane can help designers determine the misalignment tolerance of the selected transfer coil topology, which is also important.

This paper presents the results of measurements performed with two different coil topologies: classic square planar coils and the directional DD planar coils. Both coil topologies had a 100 × 100 mm footprint. The square planar coil had a larger coupling coefficient compared to the directional DD coil. This was due mainly to the larger coil size. The outer dimensions of the DD coil loop measured 100 × 50 mm compared to the outer dimensions of the planar square coil, which had the dimensions 100 × 100 mm. Therefore, for larger air gaps, the DD coil needs a larger area compared to the square coil.

When comparing the *x*-*y* plane coupling coefficient measurements presented in Figure 12, the square planar coil had a symmetrical drop in the coupling coefficient. On the other hand, the DD coil does not have a symmetrical drop in the coupling coefficient, which is presented in Figure 15. In the case of our measurements, the coils had better tolerance on the *x*-axis compared to on the *y*-axis. This was due to the directional magnetic field, which was aligned with the *y*-axis.

The last measurement was performed with one DD coil and one square planar coil, and only the *x*-*y* plane measurements were performed. The results were presented in Figure 17. When perfectly aligned, the coupling coefficient between the DD and square coil was zero. The coupling coefficient increased when the coils were misaligned in both the *x* and *y* directions. The coupling coefficient remained zero when the coils were misaligned along the *x*-axis and when the *y* misalignment was zero. This means that wireless transfer using a DD coil and a square coil is only possible when the square coil is above one of the two rectangular D coils of the DD coil.

## 7. Conclusions

The article presents the design, manufacture, and calibration of a computer-controlled platform that can serve as a tool for the development and implementation of magnetic components in IPT systems. The platform enables the automated measurement and evaluation of the coupling coefficient and the mutual inductance between the transmitter and the receiver coil of the IPT system. The platform consists of three parts: the 3D positioning mechanism, the inductance measurement circuit, and the computer application for measurement and control. The 3D positioning mechanism is used to position the coils in 3D space. The inductance measurement circuit is used to measure the self and mutual inductance between the transfer coils. The computer application is used to communicate with both systems. The functions include the automatic positioning of the coils in the 3D space, the starting of the inductance measurement, and the logging and visualisation of the measurement.

The platform can be used to measure the coupling coefficient in two different modes: coupling coefficient measurements in relation to the *z* distance between the coils and the coupling coefficient measurement in the *x*-*y* plane. Coupling coefficient measurements in *z* direction can be used to determine initial coupling coefficient between the transmitter and the receiver coil at the fixed distance. The coupling coefficient measurement in the *x*-*y* plane is especially important because it reflects in the IPT system’s tolerance to misalignment. Therefore, this platform can be used to test and measure the misalignment tolerance of the manufactured coils and to test new methods and coils with better misalignment tolerance, thus increasing the efficiency of the IPT system when the coils are not perfectly aligned. The measurement data can be approximated using polynomial equations and then used for the control and optimisation of the control algorithms, which are dependent on the coupling coefficient between the transmitter and the receiver coil.

The results obtained from measurement platform can be used during the development of IPT system for electric vehicle charging. The impact of horizontal misalignment between the transmitter and receiver coil can help to determine most suitable coil structure to achieve the required robustness and high transfer efficiency.

## Figures and Tables

**Table 1:** Resistor configurations.

Configuration	*R_1_*_,*i*_	*R_2_*_,*i*_
1	100 Ω	100 Ω
2	1000 Ω	1000 Ω
3	5000 Ω	5000 Ω

## Data Availability

Data are contained within the article.
